# Identification of SARS-CoV-2 Vaccine Epitopes Predicted to Induce Long-Term Population-Scale Immunity

**DOI:** 10.1016/j.xcrm.2020.100036

**Published:** 2020-06-08

**Authors:** Mark Yarmarkovich, John M. Warrington, Alvin Farrel, John M. Maris

**Affiliations:** 1 Division of Oncology and Center for Childhood Cancer Research, Children’s Hospital of Philadelphia, Philadelphia, PA 19104, USA; 2 Department of Biomedical and Health Informatics, Children’s Hospital of Philadelphia, Philadelphia, PA 19104, USA; 3 Perelman School of Medicine at the University of Pennsylvania, Philadelphia, PA 19104, USA

**Keywords:** COVID-19, SARS-CoV-2, coronavirus, vaccine, DNA vaccine, RNA vaccine, MHC, HLA, epitope, immunogenicity

## Abstract

Here we propose a SARS-CoV-2 vaccine design concept based on identification of highly conserved regions of the viral genome and newly acquired adaptations, both predicted to generate epitopes presented on major histocompatibility complex (MHC) class I and II across the vast majority of the population. We further prioritize genomic regions that generate highly dissimilar peptides from the human proteome and are also predicted to produce B cell epitopes. We propose sixty-five 33-mer peptide sequences, a subset of which can be tested using DNA or mRNA delivery strategies. These include peptides that are contained within evolutionarily divergent regions of the spike protein reported to increase infectivity through increased binding to the ACE2 receptor and within a newly evolved furin cleavage site thought to increase membrane fusion. Validation and implementation of this vaccine concept could specifically target specific vulnerabilities of SARS-CoV-2 and should engage a robust adaptive immune response in the vast majority of the population.

## Highlights

Selecting optimal epitopes is essential for vaccine safety and efficacy
We report 65 vaccine peptides predicted to drive long-term immunity in most people
Epitopes contain domains conserved in 15 coronaviruses and newly evolved SARS2 regions
Epitopes can be used to generate B and/or T cell vaccines (RNA and DNA)

## Graphical Abstract

**Figure:**
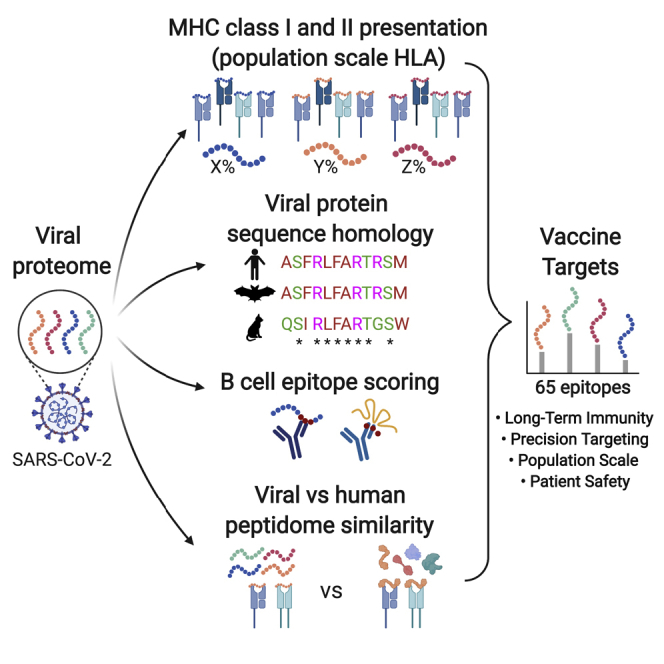

## Teaser

Yarmarkovich et al. report SARS-CoV-2 peptides for use in multi-epitope vaccines. These peptides are predicted to activate CD4 and CD8 T cells, are highly dissimilar from the self-proteome, and are conserved across 15 related coronaviruses. Presented epitopes are expected to drive long-term immunity in the majority of the population.

## Introduction

The current SARS-CoV-2 pandemic has precipitated an urgent need for a safe and effective vaccine to be developed and deployed in a highly accelerated time frame as compared with standard vaccine development processes.^1^ Upfront selection of epitopes most likely to induce a safe and effective immune response can accelerate these efforts. Optimally designed vaccines maximize immunogenicity toward regions of proteins that contribute most to protective immunity, while minimizing the antigenic load contributed by unnecessary protein domains that may result in autoimmunity, reactogenicity, or even enhanced infectivity. Here we present an immunogenicity map of SARS-CoV-2 generated to inform vaccine design based on analyses across five parameters: (1) stimulation of CD4 and CD8 T cells; (2) immunogenicity across the majority of human histocompatability leukocyte antigen (HLA) alleles; (3) targeting both evolutionarily conserved regions and newly divergent regions of the virus that increase infectivity; (4) targeting linear and conformational B cell epitopes; and (5) targeting viral regions with the highest degree of dissimilarity to the self-immunopeptidome, such as to maximize safety and immunogenicity. We present a list of SARS-CoV-2 minigenes and propose their use in multivalent vaccine constructs that should generate T and/or B cell epitopes that can be delivered by scalable manufacturing techniques such as DNA or nucleoside mRNA.

SARS-CoV-2 is the third coronavirus in the past two decades to acquire infectivity in humans and result in regional epidemics, and the first to cause a worldwide pandemic. The spike (S) glycoprotein of coronaviruses mediates host cell entry and dictates species tropism, with the SARS-CoV-2 S protein reported to bind its target protein angiotensin I converting enzyme 2 (ACE2) with 10- to 20-fold higher affinity than SARS-CoV in humans.^2^^,^^3^ In addition, insertion of a novel protease cleavage site^4^ is predicted to confer increased virulence by facilitating the cleavage necessary to expose the fusion peptide that initiates membrane fusion, enabling a crucial step of viral entry into host cells.^5^^,^^6^ It is now clear that coronavirus disease 2019 (COVID-19) results when SARS-CoV-2 infects type II pneumocytes lining the pulmonary alveoli that co-express ACE2 and the transmembrane serine protease 2 (TMPRSS2)^7^, likely impairing release of surfactants that maintain surface tension. This impairment hinders the ability to prevent accumulation of fluid, ultimately resulting in acute respiratory distress syndrome.^8^^,^^9^ The immune response of convalescent COVID-19 patients consists of antibody-secreting cells releasing IgG and IgM antibodies, increased follicular helper T cells, and activated CD4 and CD8 T cells,^10^ suggesting that a broad humoral and T cell-driven immune response mediates the clearance of infection, and that vaccination strategies directed at multiple arms of the immune response can be effective. The large size of the SARS-CoV-2 (∼30 kb) suggests that selection of optimal epitopes and reduction of unnecessary antigenic load for vaccination may be essential for safety and efficacy.

Rapid deployment of antibody-based vaccination against SARS-CoV-2 raises the concern of accelerating infectivity through antibody-dependent enhancement (ADE), the facilitated viral entry into host cells mediated by subneutralizing antibodies (those capable of binding viral particles, but not neutralizing them).^11^ ADE mechanisms have been described with other members of the *Coronaviridae* family.^12^^,^^13^ It has already been suggested that some of the heterogeneity in COVID-19 cases may be caused by ADE from prior infection from other viruses in the coronavirus family.^14^

Although the immunogenicity map presented in this study can be used to inform multiple modalities of vaccine development, we present peptide sequences that are expected to be safe and immunogenic for use in T cell-based vaccination, and highlight B cell epitopes derived from peptides within the regions of the S protein involved in infectivity that we expect will minimize the risk for ADE. Because it has been shown that T helper (Th) cell responses are essential in humoral immune memory response,^15^^,^^16^ we anticipate that the T cell epitopes generated from the peptide sequences presented here will aid the activation of CD4 T cells to drive memory B cell formation and somatic hypermutation when paired with matched B cell epitopes.

The potential of epitope-based vaccines to induce a cytolytic T cell response and drive memory B cell formation is complicated by the diversity of HLA alleles across the human population. The HLA locus is the most polymorphic region of the human genome, resulting in differential presentation of antigens to the immune system in each individual. Therefore, individual epitopes may be presented in a mutually exclusive manner across individuals, confounding the ability to immunize a population with broadly presented antigens. Whereas T cell receptors (TCRs) recognize linearized peptides anchored in the major histocompatibility complex (MHC) groove, B cell receptors (BCRs) can recognize both linear and conformational epitopes, and are therefore difficult to predict without prior knowledge of a protein structure. Here we describe an approach for prioritizing viral epitopes derived from a prioritized list of 33-mer peptides predicted to safely target the vulnerabilities of SARS-CoV-2, generate highly immunogenic epitopes on both MHC class I and II in the vast majority of the population, and maximize the likelihood that these peptides will drive an adaptive memory response.

## Results

**Figure 1:**
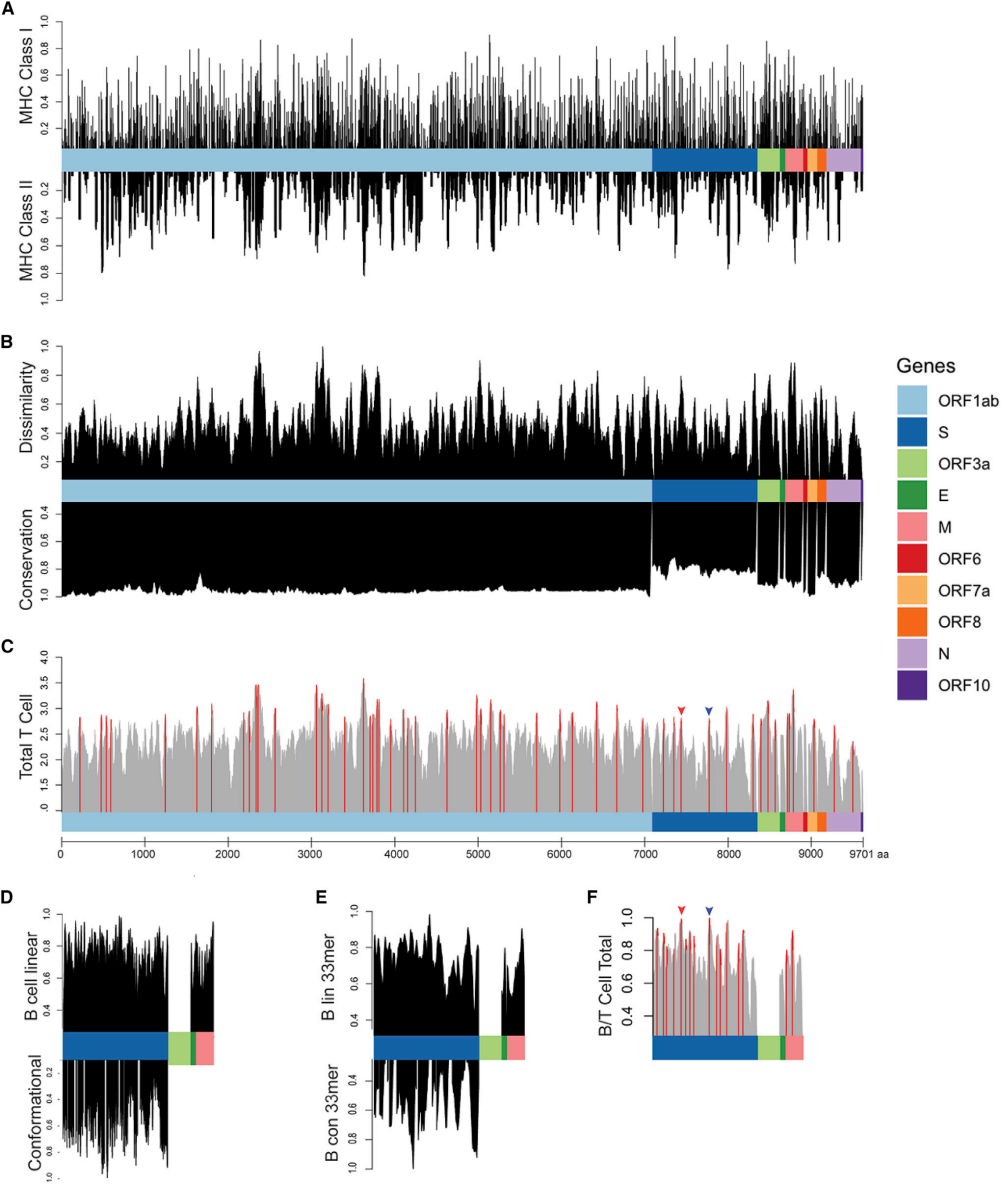
Epitope Scoring along SARS-CoV-2 Proteome (A) HLA presentation of 33-mers across viral proteome. Representation of MHC class I presentation (top) and MHC class II presentation (bottom) reported as frequency of the population predicted to present peptides derived from each region of the viral proteome. (B) Scoring of each epitope derived from the 33-mers along the length of the proteome as compared with the epitopes derived from the normal human proteome presented across 84 HLA alleles, reported as normalized scores in which the highest scoring epitopes are maximally dissimilar to self-peptides derived from normal proteins (top). Scoring for genomic conservation against 15 cross-species coronaviruses and 727 human sequences, with highest scoring regions conserved across human and other mammalian coronaviruses (bottom). (C) Combined epitope score reported as sum of four above parameters (local maximum for epitopes with 90^th^ percentile total score in red). (D) Scoring of B cell epitopes for each amino acid for linear epitopes for Spike, Envelope, and Matrix proteins (top) and conformational epitopes in Spike protein (bottom). (E) Combined scoring of 33-mer epitopes as described in (D). (F) Combined B and T cell epitope scoring in Spike, Envelope, and Matrix proteins. Receptor binding domain epitope highlighted with red arrow and epitope containing furin cleavage site highlighted with blue arrow (Figure 2).

We applied our recently published methods for scoring population-scale HLA presentation of all 9-mer peptides along the length of individual oncoproteins in human cancer to analyze the population-scale HLA presentation of peptides derived from all 10 SARS-CoV-2 genes across 84 class I HLA alleles,^17^ representing 99.4% of the population as calculated based on allele frequencies reported in the Bone Marrow Registry.^18^ A total of 6,098 SARS-CoV-2-derived peptides were predicted to bind to no HLA class I alleles, and thus we consider them immunogenically silent. In contrast, 3,524 SARS-CoV-2 epitopes were predicted to generate strong binders with least one HLA class I allele. Indeed, peptide FVNEFYAYL was predicted to bind 30 HLA alleles, representing 90.2% of the US population (Figure 1A, top; Table S1).

We next tested various peptide sequence lengths to maximize HLA presentation on multiple alleles within a single k-mer, finding that 33 amino acids generated maximal population-scale HLA presentation. We show that 99.7% of all 9,303 possible 33-mers are predicted to generate at least one HLA class I epitope, and propose that expression and presentation of these 33-mers in dendritic cells is expected to induce an immune response across a significant proportion of the population.^19^^,^^20^ We identified viral regions predicted to generate epitopes that would present across the majority of the population, highlighting a single 33-mer ISNSWLMWLIINLVQMAPISAMVRMYIFFASFY containing multiple epitopes predicted to bind 82 of the 84 HLAs alleles, suggesting that this single 33-mer can potentially induce an immune response in up to 99.4% of the population given proper antigen processing (Table S1).

Because presentation by MHC class II is necessary for robust memory B and T cell responses,^15^^,^^16^ we analyzed presentation of these viral epitopes on 36 MHC class II HLA alleles, representing 92.6% of the population (Figure 1A, bottom; Table 1; Table S1). Peptides derived from the 33-mer IAMSAFAMMFVKHKHAFLCLFLLPSLATVAYFN were predicted on 24 HLA class II alleles, representing 82.1% of the US population; peptides from the same 33-mer were predicted to be presented on 74 HLA class I alleles with a population frequency rate of 98.6%, showing that a single 33-mer can contain epitopes predicted to be presented on HLA class I and II across the majority of the population. Because HLA frequencies vary significantly by population, the frequency of individual HLA alleles can be adjusted based on specific populations using the SARs-CoV-2 immunogenicity map presented here, to customize vaccine design for groups with distinct HLA allele distributions (Table S1).

**Table 1:** Sample of Highest Scoring Viral Epitopes Suggested for Vaccination Based on MHC Class I Population-Scale Presentation, MHC Class II Population Presentation, Similarity Score, and Homology Score across 15 Mammal Species and 727 Human SARS-CoV-2 Gene Sequences

Gene Position	Epitope	HLA Class I Population Presentation	HLA Class I Alleles Bound	HLA Class I Binders	HLA Class II Population Presentation	HLA Class II Alleles Bound	HLA Class II Binders	Dissimilarity Score	Conservation Score	Combined T Cell Score	B Cell Total Score	B and T Cell Total Percentile
ORF1ab_3619	IAMSAFAMMFV KHKHAFLCLF LLPSLATVAYFN	98.6%	74	HLA-A: 0101, 0201, 0202, 0203, 0205, 0206, 0207, 0211, 0212, 0216, 0217, 0219, 0301, 1101, 2301, 2403, 2501, 2601, 2602, 2603, 2902, 3001, 3002, 3201, 3207, 6601, 6801, 6802, 6823, 6901, 8001 HLA-B: 0801, 0802, 0803, 1501, 1502, 1503, 1509, 1517, 3501, 3503, 3801, 4013, 4506, 4601, 4801, 5101, 5301, 5801, 5802, 7301, 8301 HLA-C: 0303, 0401, 0602, 0701, 0702, 0802, 1203, 1402, 1502	82.1%	24	HLA-DRB1: 0101, 0401, 0402, 0403, 0404, 0405, 0801, 0901, 1001, 1101, 1301, 1602 HLA-DPA10-DPB10: 103-201, 103-401, 103-402, 103-601, 201-101, 201-501, 301-402 HLA-DQA10-DQB10: 101-501, 102-602, 103-603, 501-201, 501-301	0.82	0.96	3.59	N/A	N/A
S_129	KVCEFQFCNDP FLGVYYHKNNK SWMESEFRVYS	98.5%	58	HLA-A: 0101, 0201, 0202, 0206, 0211, 0212, 0216, 0217, 0301, 0302, 1101, 2301, 2402, 2403, 2602, 3001, 3101, 3207, 6601, 6823, 6901, 8001 HLA-B: 0803, 1501, 1502, 1503, 1509, 1517, 1801, 2720, 3501, 3701, 3801, 3901, 4001, 4002, 4013, 4403, 4501, 4506, 4601, 4801, 5801, 7301 HLA-C: 0303, 0401, 0501, 0602, 0701, 0702, 0802, 1203, 1402, 1502	39.0%	9	HLA-DRB1: 0403, 1302, 0405, 0404 HLA-DPA10-DPB10: 103-201, 103-401, 103-601, 301-402 HLA-DQA10-DQB10: 102-602	0.60	0.83	2.80	1.14	91%
S_252	GDSSSGWTAG AAAYYVGYLQP RTFLLKYNENGT	95.7%	53	HLA-A: 0101, 0201, 0202, 0203, 0205, 0206, 0211, 0212, 0216, 0217, 0219, 2403, 2501, 2601, 2602, 2603, 2902, 3002, 3207, 3301, 6601, 6801, 6802, 6823, 6901, 8001 HLA-B: 0801, 0802, 0803, 1402, 1502, 1503, 1517, 3501, 4013, 4501, 4506, 5703, 5801, 8301 HLA-C: 0303, 0401, 0602, 0701, 0702, 0802, 1203, 1402, 1502	68.9%	15	HLA-DRB1: 0101, 0401, 0402, 0404, 0405, 0701, 0901, 1001, 1301, 1501, 1602 HLA-DPA10-DPB10: 103-301, 301-402 HLA-DQA10-DQB10: 102-602, 501-301	0.48	0.71	2.84	0.76	81%
S_462	KPFERDISTEIYQ AGSTPCNGVEG FNCYFPLQS	74.8%	27	A: 0206, 2402, 2403, 3207, 6601, 6802, 6823 B: 0802, 1402, 1502, 1503, 2720, 3503, 4002, 4013, 4201, 4506, 4801, 8301 C: 0401, 0702, 1203, 1402	18.7%	5	DRB1: 0701, 0801, 1101, 1602 DPA10-DPB10: 201-501	0.51	0.77	2.21	1.29	75.2%
N_305	AQFAPSASAFFG MSRIGMEVTPS GTWLTYTGAI	87.5%	40	HLA-A: 0202, 0203, 0211, 0212, 0216, 1101, 2403, 2601, 2602, 2603, 3101, 6601, 6801, 6823, 6901, 8001 HLA-B: 0803, 1502, 1503, 1517, 3501, 3503, 4402, 4403, 4506, 4801, 5301, 5703, 8301 HLA-C: 0303, 0401, 0501, 0702, 1203, 1402, 1502	6.4%	1	DRB1: 0901	0.46	0.91	2.31	N/A	N/A

Columns represent gene and position of first amino acid of 33-mer, number of HLA class I and II alleles predicted to bind at least one predicted epitope within 33-mer, list of bound alleles, the proportion of the population predicted to have at least one of these HLAs, normalized dissimilarity scores, normalized conservation scores, across the 33-mer, total T cell score, B cell score, and combined B and T cell percentile for 33-mers. Table includes S_462 in S protein containing novel receptor binding sites^21^ and N_305 containing five peptides shown to be immunogenic in IEDB. N/A, not applicable.

Next, we sought to identify the most highly conserved regions of the SARS-CoV-2 virus, positing that conserved regions are essential to viral replication and maintaining structural integrity, while non-conserved regions can tolerate mutations and result in antigens prone to immune evasion. To do this, we compared the amino acid sequence of SARS-CoV-2 with 14 closely related mammalian alpha and beta coronaviruses (human, bat, pig, and camel) from the *Coronaviridae* family (Table S2), scoring each amino acid for conservation across the viral strains. Additionally, we scored the conservation across the 727 SARS-CoV-2 genes sequences available at the time of this analysis (Table S2), equally weighing contributions from cross-species and interhuman variation (scores normalized to 0–1, with entirely conserved regions scoring 1). As expected, evolutionary divergence was greatest in the tropism-determining S protein and lowest in ORF1ab, which contains 16 proteins involved in viral replication (Figure 1B, bottom).

We then compared predicted viral MHC-presented epitopes with self-peptides presented in normal tissue on 84 HLA alleles across the entire human proteome as listed in the UniProt database, prioritizing antigens that are most dissimilar from self-peptides based on: (1) higher predicted safety based on decreased likelihood of inducing autoimmunity due to cross-reactivity with similar self-peptides presented on MHC; and (2) higher immunogenicity of dissimilar peptides based on an expected greater repertoire of antigen-specific T cells resulting from a lower degree of negative thymic selection. To analyze the similarity of the viral peptidome to human, we compared the 3,524 viral epitopes predicted to be presented on MHC against the normal human proteome on each of their MHC binding partners, testing each of 12,383 peptide/MHC pairs against the entire human proteome (85,915,364 normal peptides predicted across 84 HLA alleles). We assigned a similarity score for each peptide across all MHC peptides contained within a 33-mer, with high scoring peptides representing the highest degree of dissimilarity as compared with the space of all possible MHC epitopes derived from the normal proteome and a score of 0 representing an identical match in the human proteome (STAR Methods; Figure 1B, bottom; Table S1). We find regions of the viral proteome that are identical or highly similar to portions of the normal human proteome predicted to be presented on MHC, suggesting that an immune response mounted against these viral epitopes could result in an autoimmune response, while other high-scoring regions are highly dissimilar from self and expected to generate antigens with minimal likelihood of cross-reactivity (Table S1).

To assign an overall score for putative T cell antigens, we normalized each of our four scoring parameters (represented in Figures 1A and 1B) between 0 and 1 and summed each metric to obtain a final 33-mer peptide score, highlighting the local maxima of potentially generated epitopes scoring in the 90^th^ percentile (55 top scoring T cell peptides) across 10 SARS-CoV-2 genes as peptide sequences for vaccination (Figure 1C; Table S3).

Finally, we sought to characterize B cell epitopes, assessing linear epitopes in S, matrix (M), and envelope (E) proteins that are exposed and expected to be accessible to antibodies; we also characterized conformational epitopes in the S protein for which structural data are available using BepiPred 2.0 and DiscoTope 2.0.^22^^,^^23^ We found a strong concordance between linear and conformational epitope scores (p < 2e^−16^). Next, we performed an agnostic scoring of individual amino acid residues in S, M, and E proteins (Figure 1D), and then used these scores to generate scores for 33-mer peptides along the length of the protein (Figure 1E). The 33-mer VGGNYNYLYRLFRKSNLKPFERDISTEIYQAGS derived from S protein at position 445 ranked the highest based on combined linear and conformational B cell epitope scoring. We combined T cell epitope scores calculated above with available B cell epitope scores derived from the S, M, and E genes, providing a list of 65 peptides predicted to stimulate both humoral and cellular adaptive immunity (Figure 1F; Table S5).

To estimate the accuracy of our predictions, we compared the 65 unique 33-mer peptides presented in Table S5 with 92 epitopes derived from the first SARS virus (SARS-CoV) in the Immune Epitope Database (IEDB; https://www.iedb.org/home_v3.php) shown to elicit T cell responses. We found a significant enrichment in immunogenic peptides contained within the 65 selected SARS-CoV-2 33-mers as compared with the 33-mers not selected (p = 0.041), and find that the 33-mer AQFAPSASAFFGMSRIGMEVTPSGTWLTYTGAI derived from the N protein contains five immunogenic MHC class I and II antigens previously reported from SARS-CoV (GMSRIGMEV, MEVTPSGTWL, AQFAPSASAFFGMSRIGM, AFFGMSRIGMEVTPSGTW, and AQFAPSASAFFGMSR) within the single 33-mer (Table 1), demonstrating that epitopes selected using this analysis’s epitopes are more likely to be processed and immunogenic based on previous studies with SARS-CoV, and supporting the hypothesis that a single 33-mer is capable of generating multiple unique epitopes presented by multiple HLA alleles. We also found that a significant proportion of the peptides present within prioritized 33-mer have been predicted to bind MHC based on structural predictions.^24^

**Figure 2:**
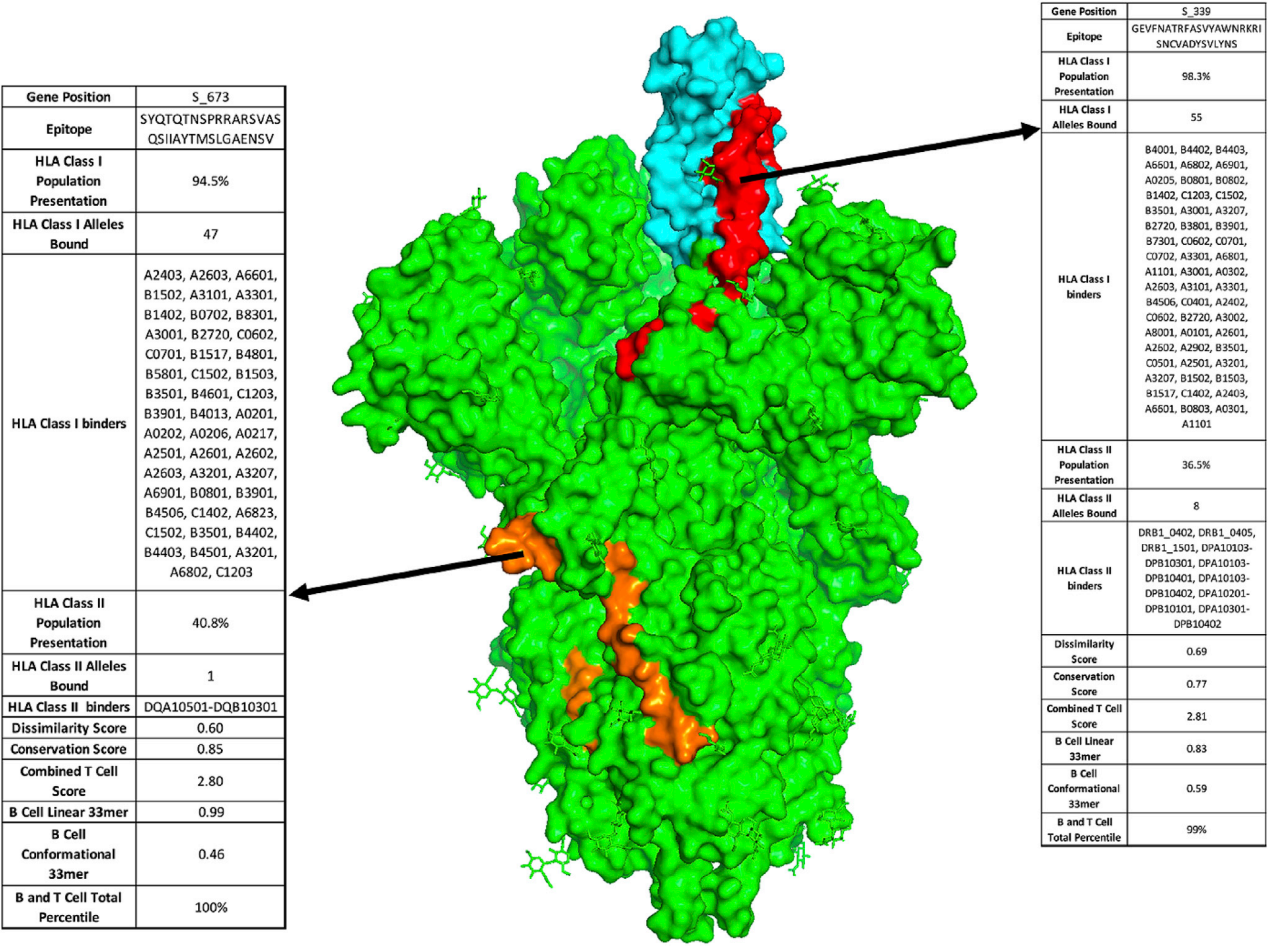
Proposed Vaccine Epitopes in SARS-CoV-2 Spike Protein Crystal structure of SARS-CoV-2 Spike protein trimer (PDB: 6VYB) with two highlighted vaccine epitopes targeting newly evolved acquired viral vulnerabilities. First, SARS-CoV-2 receptor binding domain (cyan) has up to 10-fold higher affinity binding to the ACE2 receptor as compared with previous coronaviruses. Using our analysis, we identify a high-ranking vaccine epitope (red) within the receptor binding domain. Second, SARS-CoV-2 has acquired a novel furin cleavage site RRAR, along for increased infectivity due to improved membrane fusion (epitope containing the novel furin cleavage site highlighted in orange).

In addition to prioritizing evolutionarily conserved regions, we sought to specifically target acquired vulnerabilities in SARS-CoV-2 by focusing on features of this coronavirus that have been shown to contribute to its increased infectivity. The receptor binding domain (RBD) of the SARS-CoV-2 S protein has been reported to have 10- to 20-fold higher binding affinity to ACE2.^2^ We show that viral epitope GEVFNATRFASVYAWNRKRISNCVADYSVLYNS derived from the RBD of the S protein (position 339–372) scores in the 90.9^th^ percentile of T epitopes and is the third of 1,546 epitopes scored in the S, E, and M genes for combined B and T cell epitopes, with presentation by MHC class I in 98.3% of the population (Figures 1C, 1F, and 2, red). Additionally, a recently evolved furin cleavage site has been reported in the SARS-CoV-2 virus, resulting in increased infectivity.^2^ Indeed, we find that the SYQTQTNSP**RRAR**SVASQSIIAYTMSLGAENSV peptide containing the RRAR furin cleavage site of the S protein ranked in the 90.7^th^ percentile of T cell epitopes and ranks first among the 1,546 combined B and T cell epitopes (Figures 1C, 1F, blue, and 2, orange), thereby targeting an additional evolutionary adaptation of SARS-CoV-2 with the highest overall scoring B and T cell epitope. Based on a recently published study that identified receptor binding hotspots deduced by comparing structures of ACE2 bound to the S protein from SARS-CoV-2 as compared with SARS-CoV,^21^ we searched for 33-mers containing the five acquired residues that increase S binding to ACE2, identifying KPFERDISTEIYQ**A**GSTP**C**NGVEG**FNC**YFPLQS as the highest ranked peptide sequence containing each of these residues (hotspots underlined; Table 1). Additionally, a D614G mutation in the S protein has been reported as a potentially dominant strain with increased pathogenicity.^25^^,^^26^ We thus suggest including the highest scoring 33-mer (NTSNQVAVLYQ**G**VNCTEVPVAIHADQLTPTWRV) predicted to present this mutant epitope in a vaccine construct. Finally, it is known that mRNA transcripts proximal to the 3′ end of the *Coronaviridae* family genome show higher abundance consistent with the viral replication process, with S, E, M, and N genes shown to have significantly higher translational efficiency compared with the 5′ transcripts, with the highest expression in the N gene, and consistent with the high degree of MHC presentation as described above for the five immunogenic peptides derived from a single N protein 33-mer.^27^, ^28^, ^29^ We therefore posit that viral epitopes derived from the 3′ terminus, including the S, E, M, and N genes, will have a higher representation on MHC and suggest their prioritization in a vaccine construct. Table S5 lists the highest priority viral peptides we suggest should be considered for inclusion in vaccine constructs.

## Discussion

Here we present a comprehensive immunogenicity map of the SARS-CoV-2 virus (Table S1) and propose sixty-five 33-mer peptide sequences predicted to generate B and T cell epitopes from a diverse sampling of viral domains across all 10 SARS-CoV-2 genes (Tables 1 and S5). Based on our computational algorithms, we expect that the highest scoring peptides will result in safe and immunogenic T cell epitopes, and that B cell epitopes should be evaluated for safety and efficacy using previously reported methods with validated subsets of these 65 epitopes.^12^ DNA and mRNA vaccines have been shown to be safe and effective in preclinical studies, and can be rapidly and efficiently manufactured at scale.^30^^,^^31^ Nucleoside modification of RNA has been shown to improve efficacy, which has been attributed to a reduction of RNA-induced immunogenicity.^32^ We suggest that multivalent constructs composed of the SARS-CoV-2 minigenes encoding subsets of the B and/or T cell epitopes proposed here (Tables 1, S3, S4, and S5) can be used in a DNA on mRNA vaccine for expression in antigen-presenting cells.

These epitopes can be used in tandem with a Toll-like receptor (TLR) agonist, such as tetanus toxoid or PADRE,^33^, ^34^, ^35^, ^36^ to drive activation of signals 1 and 2 in antigen-presenting cells. Constructs can be designed to contain a combination of optimal B and/or T cell epitopes, or deployed as a construct consisting of the top scoring T cell epitopes to be used in combination with the vaccines currently being developed targeting S protein in order to drive the adaptive memory response. DNA vaccine sequences can also be codon optimized to increase CpG islands, such as to increase TLR9 activation.^37^

With the third epidemic in the past two decades underway, all originating from the coronavirus family, these viruses will continue to threaten the human population, which necessitates the need for prophylactic measures against future outbreaks. The methods described here provide a rapid workflow for evaluating and prioritizing safe and immunogenic regions of a viral genome for use in vaccination. A subset of the epitopes selected here are derived from viral regions sharing a high degree of homology with other viruses in the family, and thus we expect these evolutionarily conserved regions to be essential in the infectivity and replicative life cycle across the coronavirus family. This suggests that an immune response against the aforementioned epitopes listed herein may provide more broadly protective immunity against mutated strains of SARS-CoV-2 and other coronaviruses. Additionally, we describe epitopes containing the newly acquired features of SARS-CoV-2 that confer evolutionary advantages in viral spread and infectivity. The immunogenicity map provided in Table S1 can be used to design customized multi-valent vaccines based on the HLA frequencies of specific populations. Although we suggest the use of 33-mers based on optimal MHC presentation across the population, these methods can be generalized and applied to the evaluation k-mers of various sizes depending on desired application. Because antigens may arise from the junctions between epitopes, the analyses presented here can also be used to evaluate epitope generation at the junction of specific vaccine constructs, such as to engineer linker regions that reduce the potential immunodominant epitopes elicited from irrelevant sequences.

Previous analyses of SARS-CoV-2 have predicted immunogenic epitopes based on previously reported epitopes in IEDB, sequence homology, and MHC binding predictions.^38^^,^^39^ Ahmed et al.^38^ present initial insight to potential SARS-CoV-2 epitopes by comparing previously detected epitopes derived from SARS-CoV. Grifoni et al.^39^ extended these findings by assessing sequence homology between three host species of *betacoronaviruses* and available human strains, and performing B and T cell epitope predictions. Our analysis performed at the scale of 33-mer epitopes includes the addition of dissimilarity scoring, expands the homology search across 14 species of coronavirus and 727 SARS-CoV-2 genes sequences, and covers a wider diversity of HLA coverage across the population. We searched for peptides predicted by both groups contained within our selected epitopes, finding 27 of 100 peptides reported by Ahmed et al.^38^ and 187 out of 905 peptides reported by Grifoni et al.^39^ within the sixty-five 33-mers we report. We also find up to five peptides reported by Grifoni et al.^39^ within a single 33-mer and up to 12 peptides reported by Ahmed et al.^38^ contained in the 33-mer AQFAPSASAFFGMSRIGMEVTPSGTWLTYTGAI described above. Taken together, these comparisons show a significant convergence on a subset of epitopes using agnostic analyses, while also reporting unique epitopes in each study. The finding that up to 12 epitopes from previous analyses are represented in a single 33-mer from our agnostic analysis further supports our prediction that cocktails of 33-mer epitopes can be used for population-scale vaccination.

By narrowing the pool of peptides selected for downstream screening, we expect that the analyses presented here will contribute to maximizing the efficiency of vaccine development. Antigenic burden from epitopes that do not contribute to viral protection can cause autoimmune reactions, reactogenicity, detraction from the efficacy of the vaccine, or result in ADE. We found that the vast majority of the SARS-CoV-2 virus is immunogenically silent on MHC class I and II and suggest these regions should be excluded from vaccine development. Although empirical testing is necessary to evaluate ADE, we suggest that antibodies directed at the RBD and furin cleavage sequences may mitigate ADE by blocking the processes needed to achieve membrane fusion. To avoid potential T cell cross-reactivities *a priori*, we selected maximally immunogenic epitopes with the highest degree of dissimilarity to the self-proteome with minimal potential of cross-reactivity that can lead to adverse reaction or weaken the efficacy of vaccination. In addition to the predicted safety of these epitopes (stemming from lack of potentially cross-reactive normal proteins), we expect that a greater repertoire of viral antigen-specific T cells will be present because of the absence of negative thymic selection. Although we prioritize epitopes with maximal dissimilarity from the human proteome, many other SARS-CoV-2 peptides show identical or nearly identical peptides presented on MHC derived from normal proteins. This implies that the inclusion of these highly similar epitopes in a vaccine could result in cross-reactive binding and potentially result in autoimmune responses.

Previously, it has been demonstrated that immunity acting through CD8 cells alone is sufficient in ameliorating infection, as demonstrated in studies showing that CD8-mediated vaccination is protective against influenza challenge in mice replete of antibodies and B cells,^40^ and by human CD8 cells shown to be protective across multiple influenza strains.^41^ CD8-based vaccine approaches have been shown to be particularly protective against intranasal viral transmission,^42^ suggesting that nasal protection through CD8 vaccination may be relevant to SARS-CoV-2 transmission based on recent reports of ACE2 and TMPRSS2 co-expression in nasal epithelium^7^ and clinical reports of SARS-CoV-2 infection in the olfactory bulb and symptoms of anosmia.^43^^,^^44^ Although CD8 vaccines targeting conserved antigens in influenza did not completely block infection upon challenge with virus, they effectively reduced viral replication, morbidity, and mortality.^41^^,^^42^ Taken together, these findings suggest that CD8-based immunity can be a viable strategy in quelling SARS-CoV-2. Studies demonstrating protection against multiple influenza strains imply that CD8-mediated vaccination may act more broadly than antibody responses in protecting against multiple virus family members through targeting of conserved non-structural proteins critical in the viral life cycle.

Currently, targeting CD8 epitopes has been complicated by HLA restriction of peptides and antigenic drift resulting from viral regions in which mutation is tolerable. We propose that a vaccine designed to induce CD8 responses across multiple HLA alleles covering large proportions of the population and targeting conserved regions of the virus that are highly dissimilar from the human self-peptidome can provide a safe vaccination strategy that can be rapidly tested for use alone or in combination with antibody-based vaccines in development. For example, the 33-mer ISNSWLMWLIINLVQMAPISAMVRMYIFFASFY contains epitopes predicted to be present in 99.4% of the population, scores in the 99.8^th^ percentile in dissimilarity to the human proteome, and in the 79.3^rd^ percentile in conservation. This 33-mer is derived from the most conserved region of the virus, ORF1ab, and encodes the NSP3 protein, which is critical to viral replication.^45^ These results imply that a CD8-based vaccine including such 33-mers could induce population-scale protection targeting a critical non-structural protein and circumvent safety concerns of ADE, potentially accelerating safe vaccine development.

Although the epitopes presented here are based on computational predictions (which do not account for the multiple steps involved in antigen processing and presentation), our previous validation of peptide presentation using liquid chromatography-tandem mass spectrometry (LC-MS/MS) of peptides eluted from MHC across multiple tumors showed highly significant concordance with predicted population-scale presentation.^17^ Although we expect a significant fraction of predicted antigens to be presented on MHC, binding predictions alone do not determine which antigens will elicit an immunodominant response. Although the dissimilarity scoring predicts that TCRs specific for these antigens are more likely to exist (because these TCRs are far less likely to have undergone negative thymic selection), these predictions are confounded by the TCR repertoire of a given individual and the intrinsic immunogenicity of a particular peptide, which cannot be predicted without empirical testing. Because MHC binding is a prerequisite for antigen immunogenicity, we expect that immunodominant antigens will be contained within our highest scoring epitopes. However, experimental validation will be necessary to determine the contribution of individual antigens to immunity. As a best approximation for our predictions, we show a significant enrichment of peptides previously reported in IEDB to be immunogenic in the SARS-CoV virus, contained within the 65 prioritized epitopes that we present, supporting the concept that multiple antigens derived from 33-mers can be presented across multiple HLA alleles.

We expect that the comprehensive immunogenicity map presented here can be used by the scientific community to inform the design of various vaccination modalities. We are presently designing a set of vaccine vectors and validation reagents based on these analyses that we plan to make available to the research community for testing. The 65 epitopes presented here out of the 9,303 possible 33-mers derived from SARS-CoV-2 can significantly narrow the focus of vaccine development (Table S5); these epitopes can be expressed as a single <7-kb construct, or more likely tested in various combinations delivered as a cocktail of RNA constructs encoding individual 33-mers. These vaccine constructs can be rapidly and efficiently tested for the neutralizing potential of antibodies using SARS-CoV-2 pseudo-virus,^46^ the formation of memory B cells, and induction of T cell activation using methods that we have recently developed for interrogating antigen specificities in a highly multiplexed manner.^47^ Because SARS-CoV-2 has precipitated the need to rapidly develop and deploy vaccines in pandemic situations,^48^ we suggest that this comprehensive analysis can be incorporated into a process that can be rapidly implemented when future novel viral pathogens emerge.

### Limitations of Study

The *in silico* analysis of the SARS-CoV-2 genome reported here has yet to be experimentally validated. Although it is reassuring that we demonstrate enrichment of predicted epitopes from the original SARS virus previously reported in IEDB that have been shown to be immunogenic, rigorous experimental validation of our findings is required. Computational peptide MHC binding predictions do not consider critical variables in antigen presentation, such as proteasomal degradation and peptide processing. In addition, it is unclear whether the 33-mers designed to elicit a B cell will properly fold into conformations resembling the native S protein, such as to elicit a protective antibody response. We have designed multiple DNA and mRNA constructs containing combinations of 33-mers proposed here to test hypotheses that these vaccines can elicit memory and/or cytolytic T cell response and/or protective antibodies against a SARS-S-GFP pseudovirus^46^ in HLA-A2 transgenic mice.^49^ Construct designs are available upon request.

## STAR★Methods

### Key Resources Table

**Table:** 

REAGENT or RESOURCE	SOURCE	IDENTIFIER
**Deposited Data**		

SARS-CoV-2 Immunogenicity Map	This manuscript	Table S1

**Recombinant DNA**		

Vaccine constructs	This manuscript	N/A

**Software and Algorithms**		

netMHC 4.0	Andreatta and Nielsen^50^	http://www.cbs.dtu.dk/services/NetMHC-4.0
netMHCII 2.3	Jensen et al.^51^	https://services.healthtech.dtu.dk/service.php?NetMHCII-2.3
BepiPred 2.0	Jespersen et al.^22^	https://services.healthtech.dtu.dk/service.php?BepiPred-2.0
DiscoTope 2.0	Kringelum et al.^23^	https://services.healthtech.dtu.dk/service.php?DiscoTope-2.0
shinyNAP	Yarmarkovich et al.^17^	https://www.frontiersin.org/article/10.3389/fimmu.2020.00069/full
Clustal Omega	Sievers et al.^52^	https://www.ebi.ac.uk/Tools/msa/clustalo/

### Resource Availability

#### Lead Contact

Further information and requests for resources and reagents should be directed to and will be fulfilled by the Lead Contact, John M. Maris (maris@email.chop.edu).

#### Materials Availability

Vaccine constructs and testing reagents are available from the Lead Contact, John M. Maris with a completed Materials Transfer Agreement. Please email maris@chop.edu.

#### Data and Code Availability

All raw data has been reported in paper and models are described in STAR Methods.

### Method Details

#### Population-scale HLA Class I & II Presentation

We identified potential SARS-CoV-2 epitopes by applying our recently published algorithm for scoring population-scale HLA presentation of tumor driver gene, to the SARS-CoV-2 genome (GenBank Acc#: MN908947.3).^17^ All possible 33-mer amino acid sequences covering every 9-mer peptide from the 10 SARS-CoV-2 genes were generated and we employed netMHC-4.0 to predict the binding affinities of each viral 9-mer peptide across 84 HLA class I alleles.^50^ We considered 9-mer peptides with binding affinities < 500nM putative epitopes. MHC class II binding affinities were predicted as described above across 36 HLA class II alleles population using netMHCII 2.3.^51^ All 9mers present in a 33-mer contribute to the score. 33-mer scores calculated by infering population scale hla presentation of all predicted peptides within 9-mer on class I and ii.

The frequencies of HLA class I alleles -A/B/C and HLA class II alleles -DRB1/3/4/5 were obtained from Be the Match bone marrow registry.^18^ HLA class II alleles -DQA1/DQB1 and -DPA1/DPB1 were obtained from ^53^ and ^54^, respectively.

#### Conservation Scoring

We obtained all 727 unique protein sequences categorized by each of the 10 SARS-CoV-2 genes available from the NCBI as of 25 March 2020. All sequences were aligned using Clustal Omega^52^ and each position summed for homology. In addition to human sequences, we scored each amino acid position for homology across 15 species of related coronavirus found in bats, pigs, camels, mice, and humans (SARS-CoV, SARS-CoV-2, and MERS). Each amino acid was scored up to 100% conservation. 33-mer peptides were then scored in Equation 1:

[1] $$C=\frac{\sum_{1}^{33}A_{i}-\text{Y}}{Z-Y}$$

Where C is the 33-mer conservation score, A is the conservation percentage of an amino acid position, Y is the minimum 33-mer conservation percentage sum, and Z is the maximum 33-mer conservation percentage sum. In the same way, we ranked the conservation across 274 SARS-CoV-2 amino acid sequences available at the time of this study. A final conservation score was generated by averaging the conservation scores from cross-species and interhuman variation and 33-mer peptides with the highest score were considered the most conserved.

#### Dissimilarity Scoring

3,524 viral epitopes were compared against the normal human proteome on each of their MHC binding partners, testing a total of 12, 383 peptide/MHC pairs against the entire human proteome (85,915,364 normal peptides across HLAs), assigning a similarity score for each peptide. Residues in the same position of the viral and human peptides with a perfect match, similar amino acid classification, or different polarity, were assigned scores of five, two, or negative two respectively. Similarity scores were calculated based on amino acid classification and hydrophobicity were determined using non-anchor residues on MHC (Figure S1A). The canonical TCR-interaction hotspots (residues four through six) were double weighted.^55^, ^56^, ^57^ The similarity scores generated for each viral peptide were converted to Z-scores and peptides with a p < 0.0001 were selected for comparison to viral epitopes (Figure S1B). The overall dissimilarity score for the viral peptide was then calculated using Equation 2:

[2] $$S_{Sim}=Z_{Max}-\left(Z_{Top}+{\frac{N_{Sig}}{1000}}^{\frac{\bar{Z_{Sig}}}{Z_{max}}}\right)$$

where $S_{Sim}$ is the overall dissimilarity score for the viral peptide, $Z_{Max}$ is the highest possible Z-score given a perfect sequence match to the viral peptide,$\;Z_{Top}$ is the highest Z-score from the human proteome, $N_{Sig}$ is the number of statistically significant peptides from the human proteome, and $\bar{Z_{Sig}}$ is the mean Z-score from the statistically significant peptides given a p < 0.001.

#### B cell Epitope Scoring

We used BepiPred 2.0 and DiscoTope 2.0^22^^,^^23^ to score individual amino acid residues, assessing linear epitopes in Matrix, Envelope, and Spike proteins, and conformational epitopes for Spike protein, based on published structure (PDB 6VYB). To we summed and normalized linear and conformational, using separate normalizations for proteins in which only linear predictions were available.

## Supplemental Information

Document S1. Figure S1

Table S1. Immunogenicity Map of SARS-CoV-2, Related to Figure 1 and Table 1 Combined analysis of SARS-CoV-2 for HLA class I & II binding, dissimilarity scoring, and conservation scoring for peptides beginning at each position in the viral proteome. HLA binding reported as percent rank of epitopes and 33mers beginning at each position, with a ≤ 0.5 threshold for listed binders (strong binders). Dissimilarity, conservation and B cell (linear and conformational) scores listed for 33mers starting at each peptide.

Table S2. Sequences Used for Conservation Scoring, Related to Figure 1 and Table 1 Sequences for 14 alpha and beta coronavirus genes and 727 SARS-CoV-2 genes.

Table S3. Top Scoring SARS-CoV-2 T Cell 33-mers, Related to Figure 1 and Table 1 Fifty-five highest scoring 33-mer peptides based on a combination of HLA class I, class II, dissimilarity, and homology scoring (contains peptides found to be B cell epitopes as well).

Table S4. Top Scoring SARS-CoV-2 B and T Cell 33-mers, Related to Table 1 and Figure 1 Seventeen highest scoring 33-mer peptides based on combined B and T cell scoring.

Table S5. Prioritized List of Sixty-five 33-mer Peptide Sequences Enriched for Population-Scale Immunity, Related to Figure 1 and Table 1

Document S2. Article plus Supplemental Information
