# Supplementary material for: Identification of SARS-CoV-2 Vaccine Epitopes Predicted to Induce Long-Term Population-Scale Immunity
Source: Cell Rep Med. 2020 Jun 8;1(3):100036. doi: 10.1016/j.xcrm.2020.100036 (PMC7276303; doi:10.1016/j.xcrm.2020.100036)
Supplement: Document S1. Figure S1 [file mmc1.pdf]

Cell Reports Medicine, Volume *1*

Supplemental Information

Identification of SARS-CoV-2 Vaccine Epitopes Predicted to Induce Long-Term Population-Scale Immunity

Mark Yarmarkovich, John M. Warrington, Alvin Farrel, and John M. Maris

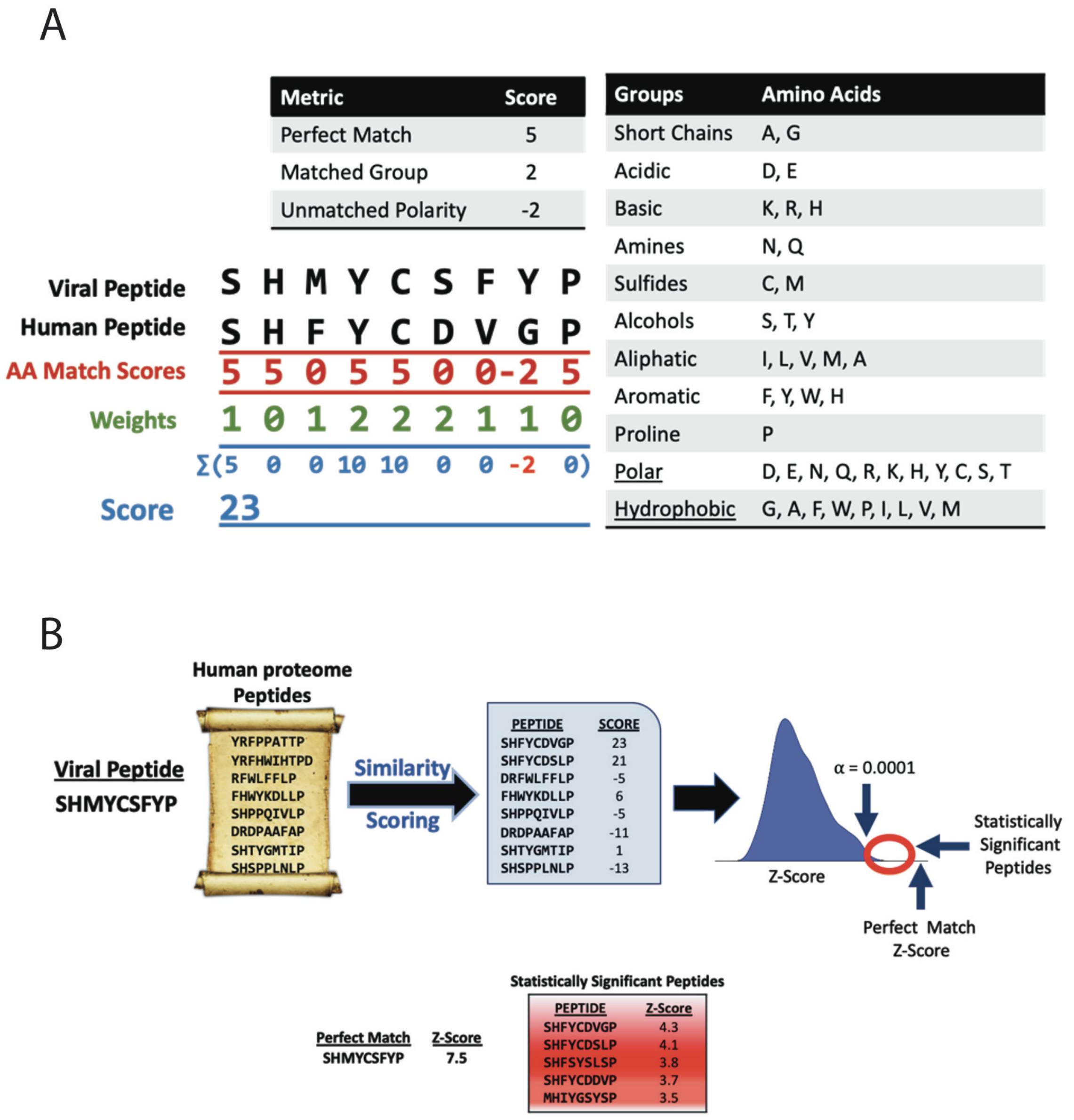

$$Disimilarity\ Score = Z_{Max} - \left(Z_{Top} + \frac{N_{Sig}^{\frac{\overline{Z_{Sig}}}{Z_{max}}}}{1000} \right)$$

$$Disimilarity\ Score = 7.5 - \left(4.3 + \frac{6^{\frac{3.9}{7.5}}}{1000} \right)$$

$$Disimilarity\ Score = 3.13$$

Figure S1. Dissimilarity Scoring, related to STAR Methods Dissimilarity Scoring. A) 3,524 viral epitopes (12,383 total peptide/MHC pairs) were compared against the normal human proteome. Non-anchor residues were used to calculate similarity scores based on amino acid classifications as described in methods. Residues in the same position of the viral and human peptides with a perfect match, similar amino acid classification, or different polarity, were assigned scores of five, two, or negative two, respectively. B) Each viral peptide/HLA pair was compared against the set of normal peptides presented on the same MHC. Dissimilarity score for each viral peptide was calculated by comparing against the most similar group of peptides with p < 0.0001 and reported as the difference in Z-scores between the viral peptide and closest-scoring peptides.
